# Childhood socioeconomic status and executive function in childhood and beyond

**DOI:** 10.1371/journal.pone.0202964

**Published:** 2018-08-24

**Authors:** Briana S. Last, Gwen M. Lawson, Kaitlyn Breiner, Laurence Steinberg, Martha J. Farah

**Affiliations:** 1 Department of Psychology, The University of Pennsylvania, Philadelphia, Pennsylvania, United States of America; 2 Department of Psychology, The University of California—Los Angeles, Los Angeles, California, United States of America; 3 Department of Psychology, Temple University, Philadelphia, Pennsylvania, United States of America; University of Zurich, SWITZERLAND

## Abstract

Socioeconomic status (SES) predicts health, wellbeing, and cognitive ability, including executive function (EF). A body of recent work has shown that childhood SES is positively related to EF, but it is not known whether this disparity grows, diminishes or holds steady over development, from childhood through adulthood. We examined the association between childhood SES and EF in a sample ranging from 9–25 years of age, with six canonical EF tasks. Analyzing all of the tasks together and in functionally defined groups, we found positive relations between SES and EF, and the relations did not vary by age. Analyzing the tasks separately, SES was positively associated with performance in some but not all EF measures, depending on the covariates used, again without varying by age. These results add to a growing body of evidence that childhood SES is associated with EF abilities, and contribute novel evidence concerning the persistence of this association into early adulthood.

## Introduction

Childhood socioeconomic status (SES) is predictive of performance on a range of neurocognitive measures [1,2], including executive function (EF) tasks [3]. EF is a broad and multifaceted construct, referring to a range of abilities from abstract thought to decision-making. There is an equally wide variety of tasks that neuropsychologists use to measure EF in children and adults. Given that EF abilities predict important life outcomes [4–6], and partially mediate the relation between SES and academic achievement in early [7] and later [8] childhood, the study of SES and EF is of practical as well as scientific interest.

From a practical point of view, if the effects of childhood SES on EF persist beyond childhood, these effects are even more consequential than if they are childhood-limited. Studies have examined the relation of childhood SES and EF across childhood and adolescence and have found a persistent relation that does not diminish over time. Between the ages of 4 and 6, Hughes et al. [7] found an SES disparity in EF and improvement in EF with age, with the SES disparity remaining constant across these young ages. In studies of EF development from early to middle childhood [9] and specifically working memory development from early through mid-adolescence [10] the SES disparity in EF was consistent across these ages. What remains unknown is whether childhood SES effects on EF hold steady into adulthood when EF development is finally complete. If childhood SES disparities in EF are larger at older ages, this would increase the importance of understanding and addressing these disparities; if they are smaller for older individuals, they would be less impactful in the long run.

In addition to the pragmatic question of the impact of childhood SES on adult EF, a comparison of SES disparities at different ages is also scientifically informative, as it puts constraints on the mechanisms by which SES and EF are associated. For example, if the SES gap reflects a slower process of EF development, whereby lower SES individuals eventually “catch up,” then we would expect the SES disparity in EF to narrow or close, and thus to observe smaller SES disparities among older individuals. In contrast, if ongoing effects of the low SES environment accumulate, or if the childhood gap initiates a cascade of cognitive difficulties, by which earlier EF disadvantage impedes opportunities for later EF development [11], then we would expect larger disparities among older individuals. Finally, if childhood SES is associated with EF ability through early life processes that have lasting consequences (“programming”) or innate differences, then we would not expect to observe differences in the size of the SES disparity from middle childhood through adulthood.

The present study is the first to address these practical and scientific questions. In order to do so, a study must examine EF at ages spanning childhood through adulthood and, in order to permit the direct comparison of the childhood SES-EF relation across these ages, the study must employ a common set of EF tasks which are nevertheless age-appropriate, and a common SES measure. Regarding the tasks used here, six well-known EF tasks were administered to healthy normal subjects as part of a larger study aimed at studying, cross-sectionally, the transition in cognitive control over affective processing from childhood to adulthood [12]. Subjects ranged in age from 9–25 years old, all performing the same set of tasks. Regarding SES, subjects came from a diverse range of socioeconomic backgrounds. Crucially, the SES of all subjects, regardless of age, was estimated as their family SES at the age of 8–10 years old. Subjects or their parents (depending on subject age at testing) retrospectively reported parents’ educational attainments and occupations at this time. These aspects of SES are typically recalled with accuracy even years later. To our knowledge, most studies of the association between SES and EF in youth measure concurrent SES as opposed to measuring childhood SES anchored to the same age. Our study is unique in measuring SES in a standard way at one age to compare across subjects of different ages.

In analyzing the data, the relation between SES and all EF tasks together is examined, as well as results from working memory tasks together and inhibitory control tasks together, and finally results from individual EF tasks. In the US race and ethnicity are related to SES in complex ways, with measures of SES not entirely independent of race and ethnicity. For example, a given number of years of parental education may reflect different levels of education attained, because under-resourced minority neighborhood schools provide less effective teaching. In addition, at similar levels of education and occupation in the US, there is a substantial wealth gap between races [13]. Finally, race and ethnicity are related to sources of stress and hardship quite apart from socioeconomic factors, which affect cognitive performance [14]. Many studies of SES and cognition do not include race or ethnicity among their covariates. However, given the complex relations between these constructs, we report all analyses with and without race and ethnicity. If similar effects of the SES measure are obtained with both analyses, they can more confidently be interpreted as reflecting the effect of SES per se.

## Participants

The sample included 185 participants between the ages of 9 and 25 (mean age 17.62 years, 4.42 years). This sample was drawn from a larger study on emotional influences on cognitive control (*N* = 276) (see [12], for details of recruitment criteria and procedures), to include just the participants for whom age 8-10-year parental SES information was available. As can be seen in Table 1, slightly more than half of the participants were female, and the sample was diverse in terms of race, ethnicity and socioeconomic status.

**Table 1:** Sample demographics (*N* = 185 subjects).

VARIABLE	*N* (%)	MEAN(*SD*)
**Age at assessment**		17.62(4.42)
**Female**	105(56.76)	
**Race/Ethnicity**		
Asian/Asian American	26(14.05)	
Black or African American	38(20.54)	
Caucasian/White	71(38.38)	
Hispanic/Latino(/a)	30(16.22)	
Other	19(10.27)	
**Parental Education** (where 1 = less than a high school degree; 5 = some education beyond college)		3.47(1.38)
**Parental Occupational Status—**Hollingshead categories (where 1 = farm laborers, menial service workers, students, housewives, dependent on welfare, no regular occupation and 9 = higher executive, proprietor of large businesses, major professional)		6.44(1.89)

## Procedure

Data collection occurred at two different sites, the University of California—Los Angeles (UCLA) and Weill Cornell Medical College. Participants were recruited from the community and completed two testing sessions in two visits, which included questionnaires on behavior and demographic information, computer-based testing, and a scanning session. Adults provided informed written consent, and minors provided assent. The institutional review board at each site approved the study. The task battery was administered on a desktop computer. Prior to the start of each task, an experimenter read and explained the instructions, gave a demonstration, and provided practice with the tasks.

## Measures

### Childhood SES

Participants (for participants age 18 or older) or parents of participants (for participants under age 18) reported on the educational attainment and occupation of each primary caregiver at the time when the participant was 8–10 years old. Income information was not requested because it is less reliably recalled than educational attainment and occupation. Parental educational attainment was reported in six categories: 1 = less than high school, 2 = high school, 3 = 2-year degree, 4 = 4-year degree, 5 = master’s degree, 6 = doctorate/professional degree. When parental education information was provided for two caregivers, parental education was computed as an average of both caregivers. Parental occupational prestige was coded using Hollingshead’s nine-point scale [15]. In order to assess the reliability of this system with these data, two raters (one undergraduate student and one graduate student) coded the retrospective occupation information. Using these two sets of scores, reliability was calculated by using a two-way random intraclass coefficient, i.e. ICC (2,1). The reliability was as follows: ICC (2,1): 0.84 for parent 1; 0.86 for parent 2; and 0.85 for both parents (using scores for each parent as independent observations). These values indicate excellent reliability [16]. We then calculated a retrospective SES composite score by averaging the z-standardized parental education and parental occupation variables. This variable was mean-centered for use in analyses. The retrospective non-normalized SES characteristics are shown in Table 1.

### Behavioral battery

Six commonly used EF tasks were administered. Protocols for the versions used here are available at dx.doi.org/10.17504/protocols.io.ri9d4h6. Relative to Miyake’s influential three-part analysis of EF, consisting of working memory, inhibitory control and attention shifting [17], most of the tasks included here primarily tap either working memory (WM) or inhibitory control (IC). The exception is the Verbal Fluency task, which is more difficult to analyze in terms of simple components of EF ability [18]. None of the measures available to us assessed attention shifting.

#### Digit span (WM)

A digit-span working memory task, similar to that in the Wechsler Scales [19], was used as a measure of auditory verbal working memory. Participants heard 14 “forward trials,” in which they were asked to recall sequences of digits (beginning with sequences of 2 digits and increasing to 8 digits) exactly as they were heard, and 12 “backward trials” in which they were asked to recall sequences of digits (beginning with 2 digits and increasing to 7 digits) backwards. The primary outcome measure was computed by averaging the number of correct forward trials and backward trials.

#### Letter working memory (WM)

In our study, we evaluated working memory in a computerized adaptation of a canonical working memory task [20], with the addition of interference. In this task, the participant sees four letters on the screen, followed by a screen displaying a target letter. The participant is then asked whether the target was among the four letters. In this version, two of the probe four letters presented had appeared in the previous trial, providing interference with recall on the present trial. An overall accuracy of working memory score is computed by averaging the number of correct responses across four trial-types (yes and no, interference and no interference).

#### Spatial working memory span (WM)

This task assesses the length of sequence of locations that can be held in working memory and immediately repeated, using a series of red squares that appear in different locations in a grid. The length of sequence increased until participants failed to correctly recall two successive trials at a given list length. Following [21], the measure of span was the length of the longest sequence the participant correctly recalled.

#### Stroop (IC)

A computerized version of the Stroop color-word task [22] was used to assess prepotent response inhibition. On each trial, the participant viewed either a color-word (e.g., “blue”) or a non-color word (e.g., “math”) and was instructed to identify with a button press the color in which the word was printed. In this version of the task, all color-word trials were incongruent, such that the color in which the word was printed did not match the semantic meaning of the word. Participants completed two 48-trial blocks. The measure of response inhibition was the proportion of correct color-word (i.e., incongruent) trials.

#### Tower of London (IC)

We used a computerized version of the Tower of London task [23], a task often used to measure planning and, when latency to first move is the focus of interest, also used to measure impulsive responding. Participants were asked to move balls from one tower to another with constraints on the order in which the balls can be moved, which requires planning multiple moves ahead. Participants were presented with five sets of four problems of varying difficulty, two classified as “easy” and two as “hard.” Following [24], latency to first move for “hard” problems was used as a measure of planning, with longer latencies indicative of more planning and less impulsivity.

#### Verbal fluency

We used two canonical assessments of verbal fluency, which assess EF through self-organized long-term memory search of phonological and semantic memory [25]. In the former, participants were given 1 minute to generate as many words as possible which either began with a specific letter (three trials in total of the letters “F”, “A”, and “S”) or were members of a category (three trials in total of the semantic categories “animals,” “fruits” and “vegetables”). Our outcome was a total verbal fluency score, which was computed by adding the number of words generated for each of the six trials.

### Statistical approach

Distributions over subjects of the dependent measures for each task were examined for departure from normality by visual inspection and using the Shapiro-Wilk test. Data were examined for outlier values that were 1.5 times the maximum and minimum values of the interquartile range of the performance data. Rather than eliminate these data points, we employed a Winsorizing procedure whereby low outliers were set to the value of the 5th percentile, and high outliers were set to the value of the 95th percentile.

Because performance in the six tasks was measured in different units, all dependent measures were standardized to put them on a common scale. Pearson correlations were computed for all task outcomes, to better understand the dependent measures and to assess their suitability for MANCOVA. Moderate correlations are recommended for MANCOVA [26], although only high correlations are problematic [27].

Our plan was to examine the SES-EF relation with differing degrees of aggregation across tasks, in each case asking whether the SES-EF relation is consistent across ages. Using MANCOVA, we began by assessing the main effect of childhood SES across all 6 EF tasks and the interaction of the SES effect with age. Using the same approach, we assessed the effect across the 3 working memory tasks and the 2 inhibitory control tasks, again testing for differences in the size of the effect at different ages. Pillai’s trace and its associated approximated F, a transformation of the test statistic which has approximately an F distribution, were the test statistics chosen for the MANCOVAs. Finally, we examined performance in each task separately, using multiple regression to test whether childhood SES had an effect on task performance and whether that effect varied with age.

In the MANCOVA, SES was coded as group variable based on a median split, and in the regressions SES was coded as a continuous variable. Other variables examined in all analyses were gender, site and two age-related measures, age and age^2^, the latter to capture possible nonlinear effects of age, specifically decelerating improvement with age. In addition, all analyses were analyzed with and without race/ethnicity as a covariate, with “Asian,” “Caucasian,” “Hispanic/Latino” and “Other” dummy coded relative to “African American.” Critically, for purposes of testing the consistency of SES effects over development, two interaction terms were also included: SES x age and SES x age^2^.

## Results

A total of 3% of the data were Winsorized to reduce extreme values. No data were eliminated. Correlations among the dependent variables are shown in S1 Table. They vary considerably, but most are moderate and none are high, thus meeting the prerequisites for MANCOVA. Table 2 shows the means and standard deviations for each EF measure, by the high and low SES groups.

**Table 2:** Descriptive statistics for each EF measure by SES group (*N* = 185).

SES Group	Digit Span (number correct)		Letter Working Memory (number correct)		Spatial Working Memory (span length)		Stroop (proportion correct)		Tower of London (milliseconds)		Verbal Fluency (total number of words)	
	*Mean*	*SD*	*Mean*	*SD*	*Mean*	*SD*	*Mean*	*SD*	*Mean*	*SD*	*Mean*	*SD*
**Low SES**	8.02	2.16	6.86	1.35	5.26	1.69	0.88	0.16	5367.11	4029.36	79.10	24.75
**High SES**	8.83	2.36	7.30	0.90	5.48	1.67	0.89	0.17	8895.64	10041.47	87.41	24.26

### Results from aggregated EF tasks

Results from the MANCOVA analyses on all six tasks together are presented in Table 3. Consistent with the previous literature [3] subjects with higher childhood SES performed better than those with lower. In addition, older subjects performed better than younger. Relevant to the stability of SES effects across development, these two significant factors did not interact (p = 0.61). The effects of age^2^, which captures the nonlinear, decelerating effect of age on EF, was significant, but also did not interact with SES (p = 0.99). Females performed better than males overall, but there was no difference between the sites.

**Table 3:** MANCOVA as a function of low and high SES (*N* = 185).

	*DF*	Pillai’s Trace	Approximated *F*	Numerator *DF*	Denominator *DF*
**High vs low SES**	1	0.12**	3.57	6	150
**Age**	1	0.41***	17.18	6	150
**Gender**	1	0.08*	2.18	6	150
**Site**	1	0.06	1.55	6	150
**Age**^**2**^	1	0.08*	2.18	6	150
**SES x Age**	1	0.03	0.75	6	150
**SES x Age**^**2**^	1	0.00	0.08	6	150

* = *p* <0.05

** = *p* < 0.01

*** = *p* < 0.001

Results were qualitatively similar when race and ethnicity were included as covariates, as shown in Table 4. High and low SES groups again differed from one another in EF task performance, and again there was a significant main effect for age and age^2^, and gender. Race/ethnicity also had a significant effect. As before, SES did not interact with age (p = 0.56) or age^2^ (p = 0.99).

**Table 4:** MANCOVA as a function of low and high SES controlling for race (*N* = 185).

	*DF*	Pillai’s Trace	Approximated *F*	Numerator *DF*	Denominator *DF*
**High vs low SES**	1	0.15***	4.20	6	146
**Age**	1	0.44***	19.48	6	146
**Gender**	1	0.09*	2.36	6	146
**Race**	4	0.39***	2.68	24	596
**Site**	1	0.05	1.15	6	146
**Age**^**2**^	1	0.09*	2.41	6	146
**SES x Age**	1	0.04	0.82	6	146
**SES x Age**^**2**^	1	0.00	0.09	6	146

* = *p* <0.05

** = *p* < 0.01

*** = *p* < 0.001

### Results from aggregated WM tasks

The MANCOVA results for the three working memory tasks are shown in Table 5. Childhood SES and age at testing affected working memory performance, but no other effects or interactions were significant, including the SES x age interaction (p = 0.19) and the SES x age^2^ interaction (p = 0.99). This pattern was unchanged when race and ethnicity were included as covariates, as shown in Table 6, with SES, age and race/ethnicity all predicting EF and neither interaction present (p = 0.16 and p = 0.96, respectively).

**Table 5:** MANCOVA of working memory measures as a function of low and high SES (*N* = 185).

	*DF*	Pillai’s Trace	Approximated *F*	Numerator *DF*	Denominator *DF*
**High vs low SES**	1	0.09***	5.78	3	169
**Age**	1	0.29***	23.05	3	169
**Gender**	1	0.02	1.22	3	169
**Site**	1	0.02	1.42	3	169
**Age**^**2**^	1	0.03	1.82	3	169
**SES x Age**	1	0.03	1.79	3	169
**SES x Age**^**2**^	1	0.00	0.19	3	169

* = *p* <0.05

** = *p* < 0.01

*** = *p* < 0.001

**Table 6:** MANCOVA of working memory measures as a function of low and high SES controlling for race (*N* = 185).

	*DF*	Pillai’s Trace	Approximated *F*	Numerator *DF*	Denominator *DF*
**High vs low SES**	1	0.11***	6.66	3	165
**Age**	1	0.33***	26.51	3	165
**Gender**	1	0.02	1.28	3	165
**Race**	4	0.26***	3.99	12	501
**Site**	1	0.02	0.84	3	165
**Age**^**2**^	1	0.04	2.09	3	165
**SES x Age**	1	0.03	1.85	3	165
**SES x Age**^**2**^	1	0.01	0.29	3	165

* = *p* <0.05

** = *p* < 0.01

*** = *p* < 0.001

### Results from aggregated IC tasks

Here again, as shown in Table 7, performance on the two inhibitory tasks was related to childhood SES, age at testing and gender. However, the effect of childhood SES did not differ as a function of age of testing, with SES interacting with neither age (p = 0.32) nor age^2^ (p = 0.98). This same pattern of SES and age effects, but no interactions, held true when race and ethnicity were covaried, as shown in Table 8.

**Table 7:** MANCOVA of inhibition/impulsivity measures as a function of low and high SES (*N* = 185).

	*DF*	Pillai’s Trace	Approximated *F*	Numerator *DF*	Denominator *DF*
**High vs low SES**	1	0.05*	3.94	2	157
**Age**	1	0.14***	13.20	2	157
**Gender**	1	0.04*	3.06	2	157
**Site**	1	0.00	0.21	2	157
**Age**^**2**^	1	0.02	1.80	2	157
**SES x Age**	1	0.01	1.14	2	157
**SES x Age**^**2**^	1	0.00	0.02	2	157

* = *p* <0.05

** = *p* < 0.01

*** = *p* < 0.001

**Table 8:** MANCOVA of inhibition/impulsivity measures as a function of low and high SES controlling for race (*N* = 185).

	*DF*	Pillai’s Trace	Approximated *F*	Numerator *DF*	Denominator *DF*
**High vs low SES**	1	0.05*	3.90	2	153
**Age**	1	0.15***	13.04	2	153
**Gender**	1	0.04	3.00	2	153
**Race**	4	0.02	0.33	8	308
**Site**	1	0.01	0.52	2	153
**Age**^**2**^	1	0.02	1.73	2	153
**SES x Age**	1	0.02	1.25	2	153
**SES x Age**^**2**^	1	0.00	0.02	2	153

* = *p* <0.05

** = *p* < 0.01

*** = *p* < 0.001

### Results from individual EF Tasks

Multiple regressions were run for the six EF tasks, as shown in Table 9. SES significantly predicted task performance on four out of the six tasks. Four out of the six tasks also showed significant improvements with age, and two showed a pattern of decelerating improvement with age as measured by their relation to age^2^. Gender was a significant predictor only for the Stroop task, with females performing better, and the effect of site was significant for one task, with UCLA subjects performing better on verbal fluency. As with the aggregate analyses, in none of the individual task analyses was there an interaction between SES and age or age^2^ (p> 0.38 in all cases), indicating no reliable change in the magnitude of the SES effect over age. When race/ethnicity is added as a covariate, the overall results are qualitatively similar, as shown in Table 10, although the number of individually significant SES effects drops to two of the six tasks. It is difficult to interpret the specific pattern of SES effects over tasks, given the absence of any obvious distinction between the tasks that do and do not show the effect and the possibility that these differences can be attributed to measurement error.

**Table 9:** Multiple regression analyses of EF measures on SES (*N* = 185).

Predictors	Digit Span	Letter Working Memory	Spatial Working Memory	Stroop	Tower of London	Verbal Fluency
	β	β	β	β	β	β
**SES**	0.26***	0.28***	0.06	0.05	0.24**	0.21***
**Age**	1.13*	1.76**	0.62	1.91***	0.46	1.28*
**Age**^**2**^	-0.76	-1.32*	-0.34	-1.51**	-0.29	-0.77
**Gender**						
Male	0.10	0.05	0.26	-0.31*	0.08	-0.04
**Site**						
UCLA	0.21	0.08	0.27	0.15	-0.19	0.42**
**SES x Age**	0.31	-0.30	0.48	0.10	-0.20	-0.34
**SES x Age**^**2**^	-0.30	0.14	-0.44	-0.07	0.27	0.36

* = *p* <0.05

** = *p* < 0.01

*** = *p* < 0.001

**Table 10:** Multiple regression analyses of EF measures on SES with interaction terms, covarying for race (*N* = 185).

Predictors	Digit Span	Letter Working Memory	Spatial Working Memory	Stroop	Tower of London	Verbal Fluency
	β	β	β	β	β	β
**SES**	0.16*	0.13	0.01	0.00	0.20*	0.12
**Age**	1.12*	1.72**	0.63	1.90**	0.51	1.27*
**Age**^**2**^	-0.80	-1.29*	-0.39	-1.49**	-0.34	-0.75
**Gender**						
** **Male	0.12	0.01	0.21	-0.29*	0.08	-0.04
**Race**						
Asian	0.77**	0.77**	0.73**	-0.10	0.26	0.37
Caucasian	0.30	0.82***	0.39	0.19	0.14	0.47*
Hispanic	-0.26	0.37	0.52*	-0.13	-0.12	0.14
Other	0.16	0.42	0.34	0.06	0.29	0.29
**Site**						
UCLA	0.12	-0.09	0.17	0.13	-0.25	0.31*
**SES x Age**	0.41	-0.11	0.56	0.13	-0.22	-0.24
**SES x Age**^**2**^	-0.36	-0.02	-0.50	-0.10	0.30	0.28

* = *p* <0.05

** = *p* < 0.01

*** = *p* < 0.001

## Discussion

The association between childhood SES and EF is important to understand, given that both constructs predict important life outcomes such as academic achievement [4,28] and many measures of adult health [6,29]. The present study extends our understanding of the relation between childhood SES and EF in several ways. First, in most previous studies SES is measured concurrently with child and adolescent task performance, despite the possibility that children’s sensitivity to environmental context varies across ages. We present the first evidence on the relation of SES, anchored to a specific age in childhood, to young people’s EF across ages.

In addition, unlike previous work that has examined the association between SES and EF during childhood or adolescence, our study mapped the SES-EF relation across a wide range of ages, spanning childhood, adolescence and young adulthood. Despite the range of ages, we were able to use a common set of measures, numbering six in total, which were feasible for all subjects. This enabled us to address the consistency of SES effects on EF across these ages.

Results from these tasks were analyzed at different levels of aggregation, with more consistent findings when tasks were aggregated according to type of EF, or simply EF overall, compared to when they were analyzed individually. Grouping all EF tasks together showed EF performance in the battery of six tasks to be associated with SES, with and without the race/ethnicity covariates. Grouping the working memory tasks and the inhibitory control tasks into separate sets of tasks similarly showed each set to be associated with SES, with and without the race/ethnicity covariates. At the individual task level, four of the six showed significant effects of SES. The trends remained the same when race/ethnicity was covaried, but only two tasks showed statistically significant effects of SES.

As expected, performance generally improved with age. At both levels of aggregation, the effects of age were highly significant, and at the individual task level this trend was significant for all but two tasks. However, despite significant effects of childhood SES and age across these many analyses, these factors did not interact. That is, the effect of childhood SES did not appear to differ across the wide range of ages at testing. This finding has implications for how and why SES and EF are associated. It suggests that the SES disparity in EF is established early in life and holds steady into adulthood. It neither grows, through accumulated experiences linked to SES, nor diminishes, as would be expected if lower SES individuals simply take longer to reach their developmental asymptote. This also suggests that the practical advantages of strong EF will remain more available to higher SES individuals, at least through young adulthood.

The strength of these conclusions is limited by the study sample size and the cross-sectional nature of the design. It is possible that a larger sample would reveal the critical interaction between SES and age in one or more of the analyses. It is also possible that measuring EF in the same subjects over a wide range of ages would reveal developmental changes in the effect of childhood SES. Such a longitudinal study, if combined with fluctuating measures of SES (in contrast to the relatively stable measures of education and occupation) could be particularly informative concerning sensitive periods of development. Pending the availability of such data, the present findings provide the most relevant evidence at hand on the stability of SES effects on EF from childhood through adulthood. It suggests that SES disparities in EF observed in childhood cannot be expected to resolve in adulthood.

## Supporting information

S1 Table Pearson correlations of performance on EF tasks (*N* = 185). (DOCX)
