# Supplementary material for: Childhood socioeconomic status and executive function in childhood and beyond
Source: PLoS One. 2018 Aug 24;13(8):e0202964. doi: 10.1371/journal.pone.0202964 (PMC6108482; doi:10.1371/journal.pone.0202964)
Supplement: S1 Table — (DOCX) [file pone.0202964.s001.docx]

**S1 Table. Pearson correlations of performance on EF tasks (*N*=185).**

|  | **Digit Span** | **Letter Working Memory** | **Spatial Working Memory** | **Stroop** | **Tower of London** | **Verbal Fluency** |
| --- | --- | --- | --- | --- | --- | --- |
| **Digit Span** | 1 |  |  |  |  |  |
| **Letter Working Memory** | 0.54*** | 1 |  |  |  |  |
| **Spatial Working Memory** | 0.37*** | 0.20** | 1 |  |  |  |
| **Stroop** | 0.34*** | 0.44*** | 0.31*** | 1 |  |  |
| **Tower of London** | 0.19 | 0.11 | 0.12 | 0.06 | 1 |  |
| **Verbal Fluency** | 0.65*** | 0.43*** | 0.34 | 0.39*** | 0.09 | 1 |

Note: * = *p* <0.05; ** = *p* < 0.01, *** = *p* < 0.001
